# High remnant-cholesterol levels increase the risk for end-stage renal disease: a nationwide, population-based, cohort study

**DOI:** 10.1186/s12944-024-02050-y

**Published:** 2024-06-04

**Authors:** Han Na Jung, Ji Hye Huh, Eun Roh, Kyung-Do Han, Jun Goo Kang, Seong Jin Lee, Sung-Hee Ihm

**Affiliations:** 1 Department of Internal Medicine, Hallym University College of Medicine, 1, Hallymdaehak-Gil, Chuncheon-Si, Gangwon-Do 24252 Republic of Korea; 2 Department of Statistics and Actuarial Science, College of Natural Sciences, Soongsil University, 369, Sangdo-Ro, Dongjak-Gu, Seoul, 06978 Republic of Korea

**Keywords:** Remnant-cholesterol, End-stage renal disease, Korean national health insurance service, Lipoprotein, Triglyceride

## Abstract

**Background:**

The effect of remnant-cholesterol (remnant-C) on incident end-stage renal disease (ESRD) has not been studied longitudinally. This retrospective cohort study evaluated the association between remnant-C and the development of ESRD in a nationwide Korean cohort.

**Methods:**

Participants in a National Health Insurance Service health examination (*n* = 3,856,985) were followed up until the onset of ESRD. The median duration of follow-up was 10.3 years. The Martin-Hopkins equation was used to determine low-density lipoprotein cholesterol (LDL-C) levels from directly measured triglyceride, high-density lipoprotein cholesterol (HDL-C), and total cholesterol levels. Remnant-C levels were determined by subtracting HDL-C and LDL-C from total cholesterol. The risk for incident ESRD was calculated for each quartile of remnant-C, adjusting for conventional risk factors such as baseline renal function, comorbidities, and total cholesterol levels.

**Results:**

ESRD developed in 11,073 (0.29%) participants. The risk for ESRD exhibited a gradual increase according to higher levels of remnant-C, with a 61% increased risk in the highest quartile than in the lowest (hazard ratio [HR] 1.61 [95% confidence interval (CI) 1.50–1.72]). The elevated risk for ESRD in the highest quartile versus the lowest quartile was more prominent in younger than in older subjects (20–29 years, HR 4.07 [95% CI 2.85–5.83]; 30–39 years, HR 2.39 [95% CI 1.83–3.13]; ≥ 70 years, HR 1.32 [95% CI 1.16–1.51]). In addition, the increased risk for ESRD related to higher remnant-C levels was greater in females than in males.

**Conclusions:**

Independent of conventional risk factors, remnant-C levels were positively associated with incident ESRD, particularly in younger populations and adult females. Reducing remnant-C levels may be a novel preventive strategy against ESRD.

**Supplementary Information:**

The online version contains supplementary material available at 10.1186/s12944-024-02050-y.

## Background

Nearly 850 million individuals worldwide are affected by chronic kidney disease (CKD), making it a serious threat to global health [1]. CKD ranked 13th in the global list of causes of death in 2016 [2], and it is expected to be the 5th leading cause of lost life years by 2040 [3]. Despite the first decrease in its prevalence in 2020, end-stage renal disease (ESRD) continues to pose a substantial economic burden, with inflation-adjusted Medicare spending > $50 billion in 2020 [4].

Atherogenic dyslipidemia is prevalent in individuals with CKD and is hallmarked by high levels of triglycerides (TG) and low levels of high-density lipoprotein cholesterol (HDL-C), with elevated levels of intermediate- and very-low-density lipoproteins (IDLs, VLDLs, respectively) and high lipoprotein (a), but no significant increase in low-density lipoprotein cholesterol (LDL-C) [5, 6]. Nevertheless, it remains unclear whether dyslipidemia directly affects renal function and which lipid components may be responsible for renal dysfunction. A prospective cohort study involving 4483 healthy males found that low HDL-C, high non-HDL-C, or high total cholesterol increased the risk for creatinine levels > 1.5 mg/dL over 14.2 years of follow-up [7]. Elevated non-HDL-C and LDL-C levels were also positively associated with progression to renal replacement therapy in patients with stage 3–5 CKD; however, high TG or low HDL-C levels were not [8]. Moreover, several prospective studies reported that none of the conventional lipid measurements could predict poor renal outcomes after adjusting for other confounding variables [9–11].

A novel lipoprotein parameter, remnant-cholesterol (remnant-C), represents the cholesterol transported by all TG-rich lipoproteins (TRLs), including chylomicron remnants, VLDLs, and IDLs [12]. Remnant-C has been identified as a novel risk factor for all-cause mortality, diabetes mellitus (DM), and cardiovascular disease, even superior to LDL-C [13–17]. The question of the link between remnant-C and CKD has been discussed in cross-sectional studies, which have reported a greater prevalence of CKD and more advanced disease stages with higher remnant-C levels [18–20]. Supporting these findings, a study including 5150 patients with type 1 DM followed for a median of 8 years found that a 1 standard deviation rise in remnant-C levels related to a 51% increased risk for progression of diabetic nephropathy, defined as worsening albumin excretion or renal replacement therapy [21]. Similarly, in a five-year follow-up of 4237 individuals with type 2 DM, an 82.3% higher risk for diabetic nephropathy was observed in the highest tertile compared with the lowest tertile of remnant-C levels [22]. However, based on available information, no study to date has longitudinally evaluated the role of remnant-C in the development of ESRD in a population-based cohort. As such, this longitudinal retrospective study examined the hypothesis that remnant-C levels are independently associated with incident ESRD using nationwide health examination data.

## Methods

### Source of data

The Korean National Health Insurance Service (NHIS) database, a population-based cohort of health check-up programs in Korea, was analyzed in this study. NHIS participation is mandatory for approximately 97% of the Korean population. It provides a health examination every two years for participants > 40 years and a recommended once-yearly examination for employees ≥ 20 years. Comprehensive medical information, low attrition rates, and coverage of the entire population make the NHIS a well-established database. Previous studies have provided a detailed description of the NHIS database [23]. The current study had the approval of the Institutional Review Board (HALLYM 2021-08-002). Informed written consent was not required because the NHIS database was anonymized under strict confidentiality protocols.

### Study population

Among individuals who underwent health screening in 2009 (*n* = 10,601,274), 40% of adults ≥ 20 years of age (*n* = 423,415) were sampled. After excluding subjects with TG levels ≥ 400 mg/dL (*n* = 94,742), ESRD before baseline (*n* = 3788), missing data (*n* = 268,354), and ESRD within 1 year of baseline to avoid the potential effect of “reverse causation” (*n* = 10,546), a total of 3,856,985 individuals were included (Supplemental Fig. 1). Participants were grouped based on remnant-C quartile and followed up until the onset of ESRD or until December 31, 2020.

### Data collection and definitions of outcomes

Standardized, self-administered questionnaires were provided to participants to collect lifestyle and demographic data. Smoking categorization included nonsmokers, former smokers, or current smokers [24]. Alcohol consumption was quantified to classify participants as none, mild, or heavy drinkers [25]. Heavy drinking was defined as more than 30 g/day. Engaging in 3 or more times per week of vigorous physical activity or 5 or more times per week of moderate activity was considered regular exercise [25]. The bottom fifth of the total population was defined as low-income [25]. Anthropometric information and laboratory data were also provided by the NHIS. Participants’ blood pressure, height, weight, and waist circumference were assessed.

Lipid and glucose levels were measured from blood samples collected after an overnight fast of ≥ 8 h. The levels of TG, HDL-C, and total cholesterol were measured by an enzymatic method. Quality control of laboratory examinations was performed according to established procedures [26]. To overcome limitations of the Friedewald formula, which uses a fixed TG/cholesterol ratio to estimate VLDL cholesterol (VLDL-C), the Martin-Hopkins method was used to determine LDL-C levels [27]. The Martin-Hopkins method uses an individualized TG/VLDL-C ratio for each patient [27]. Remnant-C levels were determined using the well-validated formula of subtracting HDL-C and LDL-C from total cholesterol [28].

The primary endpoint was incident ESRD as defined by *International Classification of Diseases, 10th Revision* (ICD-10) codes [24]. Definitions of comorbidities, including hypertension [29, 30], DM [16], dyslipidemia [16, 31, 32], CKD [24, 33], and obesity [34, 35], are summarized in Supplemental Table 1. Individuals who were prescribed statins or fibrates at baseline were categorized as statin or fibrate users, respectively [16].

### Statistical analysis

Baseline characteristics are described according to each remnant-C quartile and compared across quartiles using a chi-squared test or one-way analysis of variance. Multivariate Cox proportional hazards regression was performed to determine the hazard ratio (HR) for incident ESRD in each remnant-C and other lipid quartile. ESRD risk was analyzed using three different models; model 1 was adjusted for age and sex; model 2 was further adjusted for BMI, estimated glomerular filtration rate, low income, smoking status, drinking, physical activity, DM, hypertension, and statin or fibrate use; model 3 was further adjusted for total cholesterol levels. The group in the first quartile was considered to be the reference. The cumulative incidence of ESRD was compared across the remnant-C quartiles by applying the Kaplan–Meier and log-rank tests.

Age- and sex-specific adjusted HRs (aHR) and 95% CIs for incident ESRD according to remnant-C quartile were calculated using Cox regression. Subgroup analyses were also performed with stratification according to the presence of hypertension, DM, impaired fasting glucose (IFG), obesity, CKD, dyslipidemia, statin use, or fibrate use. Differences were interpreted as statistically significant with a two-tailed *P* < 0.05. Statistical analyses were performed with R version 3.1.0 (R Foundation for Statistical Computing, Vienna, Austria) and SAS version 9.4 (SAS Institute, Cary, NC, USA).

## Results

### Baseline characteristics of participants

The final analysis included 3,856,985 participants. During a median follow-up of 10.3 years (interquartile range 10.1–10.6), 11,073 (0.29%) were newly diagnosed with ESRD. Baseline characteristics categorized by quartiles of remnant-C are summarized in Table 1. Compared with those in the lower quartiles, those in the higher quartiles were older and more frequently male. The higher quartile groups demonstrated worse metabolic characteristics than the lower quartiles, including higher blood pressure, BMI, and unfavorable glucose or lipid profiles. The proportion of participants with poor health behaviors, such as current smoking, irregular physical activity, and heavy alcohol consumption, and the proportion of comorbidities, such as hypertension, DM, dyslipidemia, and CKD, was greater in the higher quartiles.

**Table 1:** Baseline characteristics of participants categorized by remnant-C quartile

	Q1 (*n* = 965813)	Q2 (*n* = 964638)	Q3 (*n* = 963878)	Q4 (*n* = 962656)	*P* value
Remnant-C (mg/dL)	< 16.38	16.38–20.99	21.00–27.43	≥ 27.44	
Age (years)	42.7 ± 13.9	47.3 ± 14.2	49.5 ± 13.9	49.5 ± 13.2	< 0.001
Men (%)	368918 (38.2)	486288 (50.4)	567208 (58.9)	658432 (68.4)	< 0.001
Body mass index (kg/m^2^)	22.1 ± 2.9	23.2 ± 3.1	24.2 ± 3.1	25.1 ± 3.0	< 0.001
Waist circumference (cm)	74.9 ± 8.3	78.8 ± 8.5	81.9 ± 8.3	84.9 ± 7.9	< 0.001
Systolic blood pressure (mmHg)	117.2 ± 14.1	121.2 ± 14.7	124.1 ± 14.8	126.8 ± 14.9	< 0.001
Diastolic blood pressure (mmHg)	73.0 ± 9.5	75.4 ± 9.8	77.2 ± 9.8	79.2 ± 10.0	< 0.001
Fasting blood glucose (mg/dL)	91.6 ± 16.5	95.2 ± 19.9	98.3 ± 23.3	102.8 ± 29.4	< 0.001
Total cholesterol (mg/dL)	175.8 ± 30.0	188.1 ± 32.5	198.8 ± 33.6	215.2 ± 37.2	< 0.001
HDL-C (mg/dL)	61.6 ± 14.4	57.1 ± 13.4	53.2 ± 12.6	48.6 ± 11.7	< 0.001
LDL-C (mg/dL)	100.1 ± 27.3	112.2 ± 30.2	121.6 ± 31.2	131.5 ± 33.0	< 0.001
TG (mg/dL)	55.5 (55.4–55.5)	90.2 (90.2–90.2)	129.7 (129.7–129.8)	216.4 (216.3–216.5)	< 0.001
Remnant-C (mg/dL)	14.1 ± 1.8	18.7 ± 1.3	24.0 ± 1.8	35.0 ± 6.7	< 0.001
eGFR (mL/min/1.73 m^2^)	90.8 ± 48.7	88.0 ± 44.0	86.5 ± 44.1	85.4 ± 44.0	< 0.001
Smoking status					< 0.001
Nonsmoker	703038 (72.8)	614294 (63.7)	547129 (56.8)	458009 (47.6)	
Former smoker	101126 (10.5)	129926 (13.5)	150183 (15.6)	167697 (17.4)	
Current smoker	161649 (16.7)	220418 (22.9)	266566 (27.7)	336950 (35.0)	
Alcohol drinking					< 0.001
None	533658 (55.3)	524513 (54.4)	504568 (52.4)	447278 (46.5)	
Mild	387243 (40.1)	377826 (39.2)	380768 (39.5)	405772 (42.2)	
Heavy	44912 (4.7)	62299 (6.5)	78542 (8.2)	109606 (11.4)	
Regular exercise	175334 (18.2)	177549 (18.4)	175890 (18.3)	165221 (17.2)	< 0.001
Low income	212875 (22.0)	193974 (20.1)	179650 (18.6)	167379 (17.4)	< 0.001
Hypertension	135349 (14.0)	220287 (22.8)	285421 (29.6)	339136 (35.2)	< 0.001
Diabetes mellitus	38441 (4.0)	66228 (6.9)	93328 (9.7)	129576 (13.5)	< 0.001
Dyslipidemia	61363 (6.4)	123589 (12.8)	183315 (19.0)	308216 (32.0)	< 0.001
Chronic kidney disease	49427 (5.1)	62183 (6.5)	72030 (7.5)	78582 (8.2)	< 0.001
Statin user	40552 (4.2)	75414 (7.8)	100094 (10.4)	129233 (13.4)	< 0.001
Fibrate user	2599 (0.3)	4700 (0.5)	7393 (0.8)	17471 (1.8)	< 0.001

Data are expressed as the mean ± standard deviation or median (25–75%) for continuous variables or n (%) for categorical variables

*Remnant-C* remnant-cholesterol, *HDL-C* high-density lipoprotein cholesterol, *LDL-C* low-density lipoprotein cholesterol, *TG* triglyceride, *eGFR* estimated glomerular filtration rate

### Risk for ESRD according to lipid levels including remnant-C

**Fig. 1:**
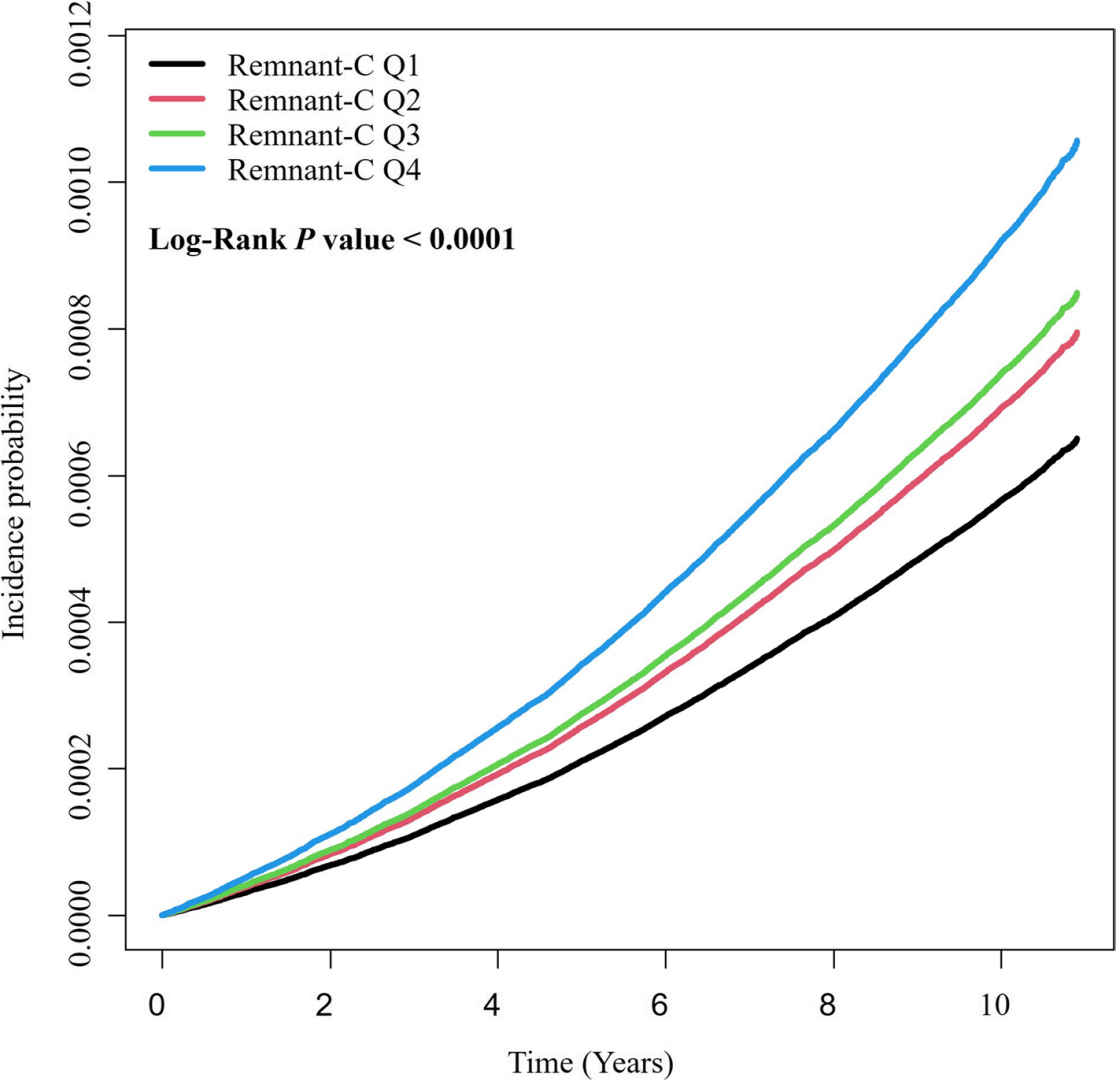
Adjusted Kaplan–Meier estimates of the cumulative incidence of ESRD by remnant-C quartile. Remnant-C, remnant-cholesterol; ESRD, end-stage renal disease

The risk for ESRD increased gradually with higher quartile of remnant-C in all adjusted models (Table 2). Relative to the first quartile group, the fourth remnant-C quartile group exhibited a 61% greater risk when adjusted for age, sex, BMI, renal function, low income, smoking and drinking status, exercise regularity, presence of DM and hypertension, use of lipid-lowering agents, and total cholesterol levels (HR 1.61 [95% confidence interval (CI) 1.50–1.72]). The adjusted Kaplan–Meier curve also demonstrated a significantly increased cumulative incidence of ESRD for subjects in higher quartiles than that for subjects in the lowest quartile, which was observed over > 10 years (*P* < 0.001) (Fig. 1).

**Table 2:** Risk for ESRD by remnant-C quartile in age subgroup

Age, years	Remnant-C	N	Events	Duration (person-years)	Incident rate (per 1000 person-years)	HRs (95% CI)		
						Model 1	Model 2	Model 3
Total	Q1	965813	1239	9823491	0.13	Reference	Reference	Reference
	Q2	964638	2298	9748549	0.24	1.38 (1.29–1.48)	1.21 (1.13–1.30)	1.22 (1.14–1.31)
	Q3	963878	3079	9719398	0.32	1.63 (1.53–1.74)	1.28 (1.20–1.37)	1.30 (1.21–1.39)
	Q4	962656	4457	9707940	0.46	2.36 (2.22–2.52)	1.56 (1.47–1.67)	1.61 (1.50–1.72)
20–29	Q1	216254	53	2228694	0.02	Reference	Reference	Reference
	Q2	119364	54	1229318	0.04	1.61 (1.10–2.35)	1.69 (1.16–2.47)	1.71 (1.17–2.50)
	Q3	75313	43	775409	0.06	1.81 (1.21–2.71)	1.90 (1.27–2.84)	1.93 (1.29–2.89)
	Q4	53160	71	546507	0.13	3.93 (2.75–5.61)	3.96 (2.77–5.66)	4.07 (2.85–5.83)
30–39	Q1	195991	69	2015669	0.03	Reference	Reference	Reference
	Q2	176645	129	1816383	0.07	1.82 (1.36–2.44)	1.75 (1.31–2.35)	1.77 (1.32–2.37)
	Q3	169358	125	1741288	0.07	1.69 (1.26–2.27)	1.54 (1.14–2.06)	1.56 (1.16–2.10)
	Q4	188626	257	1937400	0.13	2.97 (2.27–3.87)	2.32 (1.78–3.04)	2.39 (1.83–3.13)
40–49	Q1	264,292	203	2,716,479	0.07	Reference	Reference	Reference
	Q2	256404	334	2631799	0.13	1.53 (1.29–1.82)	1.31 (1.10–1.56)	1.32 (1.11–1.57)
	Q3	244386	414	2506308	0.17	1.81 (1.53–2.14)	1.34 (1.13–1.58)	1.36 (1.15–1.61)
	Q4	253134	673	2591441	0.26	2.61 (2.23–3.05)	1.56 (1.33–1.82)	1.60 (1.36–1.88)
50–59	Q1	156684	265	1601788	0.17	Reference	Reference	Reference
	Q2	204645	462	2091009	0.22	1.28 (1.10–1.49)	1.13 (0.97–1.32)	1.14 (0.98–1.33)
	Q3	227375	648	2322775	0.28	1.55 (1.34–1.79)	1.22 (1.05–1.40)	1.23 (1.07–1.42)
	Q4	233784	1091	2382145	0.46	2.42 (2.11–2.76)	1.59 (1.39–1.82)	1.63 (1.42–1.87)
60–69	Q1	87196	358	866657	0.41	Reference	Reference	Reference
	Q2	132262	670	1318621	0.51	1.25 (1.10–1.42)	1.07 (0.94–1.21)	1.07 (0.94–1.22)
	Q3	157176	1026	1569479	0.65	1.62 (1.43–1.82)	1.23 (1.09–1.39)	1.25 (1.11–1.41)
	Q4	152027	1424	1515962	0.94	2.32 (2.07–2.61)	1.53 (1.36–1.72)	1.57 (1.39–1.76)
≥ 70	Q1	45396	291	394206	0.74	Reference	Reference	Reference
	Q2	75318	649	661419	0.98	1.40 (1.22–1.61)	1.19 (1.03–1.36)	1.19 (1.04–1.37)
	Q3	90270	823	804140	1.02	1.52 (1.33–1.74)	1.18 (1.03–1.35)	1.20 (1.05–1.37)
	Q4	81925	941	734485	1.28	1.96 (1.72–2.24)	1.29 (1.13–1.47)	1.32 (1.16–1.51)

*ESRD* end-stage renal disease, *remnant-C* remnant-cholesterol, *HR* hazard ratio, *CI* confidence interval

The risk of developing ESRD was compared between quartiles of each lipid parameter according to the adjustment model 3 except for total cholesterol levels (Supplemental Table 2). A gradually increasing risk of ESRD was observed with higher remnant-C and TG levels, with the highest quartile group having a 56% and 58% higher risk, respectively, compared with the lowest quartile. The top three quartiles of HDL-C had a 27–31% reduced risk of ESRD compared with the first quartile. On the other hand, the risk in the fourth quartile group of total cholesterol and LDL-C was not different compared with the first quartile.

### Risk for ESRD according to age group

Both nephropathy and dyslipidemia are more common with advancing age [36, 37]. Therefore, the risk for developing ESRD was analyzed according to remnant-C quartile for each age subgroup (Table 2). The incremental risk for ESRD associated with a higher level of remnant-C was consistent across most age groups. Although ESRD incidence increased with age, the increased ESRD risk in the higher quartiles relative to the lowest quartile was more pronounced in younger subjects. The HR (95% CI) for incident ESRD in the highest quartile versus the first quartile were 4.07 (2.85–5.83), 2.39 (1.83–3.13), 1.60 (1.36–1.88), 1.63 (1.42–1.87), 1.57 (1.39–1.76), and 1.32 (1.16–1.51) for individuals in their 20 s, 30 s, 40 s, 50 s, 60 s, and ≥ 70 years, respectively, based on adjustment model 3.

### Risk for ESRD according to sex

The risk for incident ESRD, stratified according to remnant-C quartile, was calculated for each sex (Supplemental Table 3). Both males and females exhibited an increased risk for ESRD in higher remnant-C quartiles. According to adjustment model 3, the aHR (95% CI) for the highest versus first quartile was 1.42 (1.31–1.55) for males and 1.99 (1.78–2.23) for females. The higher risk for ESRD associated with increasing remnant-C level was also evident in all age groups and was more prominent in younger participants of both sexes. Interestingly, the increase in risk from the bottom to the top remnant-C quartile was more apparent in females than in males of all ages. The risk for developing ESRD for females in the fourth quartile was 10.1 times higher for those 20–29, 5.9 times higher for those 30–39, 3.7 times higher for those 40–49, and 1.4 times higher for those ≥ 70 years of age than those in the first quartile. For males, the corresponding risks were 4.9-, 2.6-, 1.1-, and 1.4-times higher in the same age groups, respectively.

### Risk for ESRD according to risk factor subgroup

**Fig. 2:**
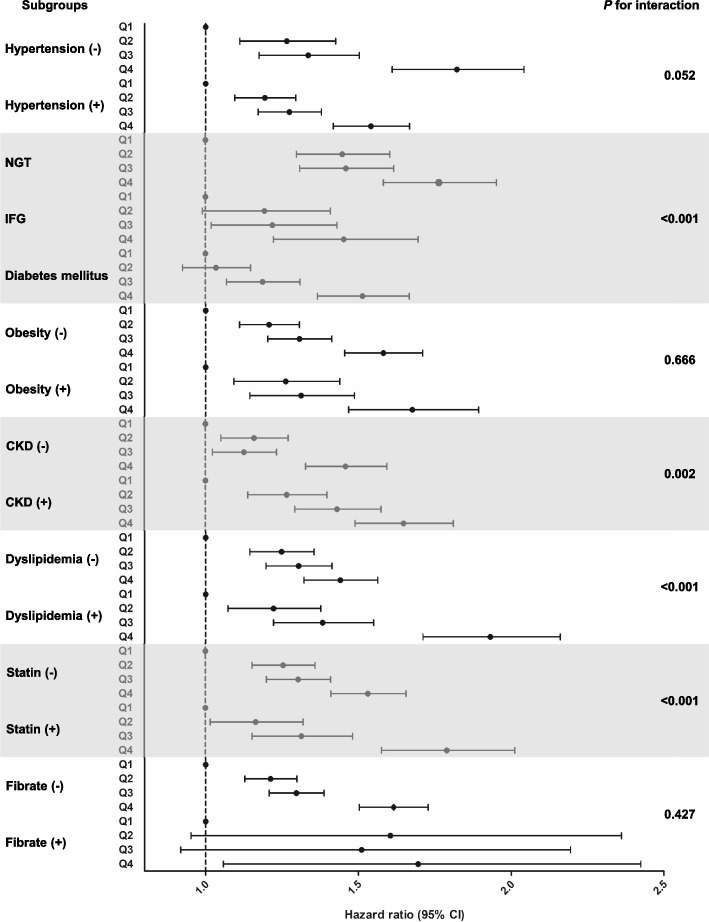
Risk for ESRD according to remnant-C quartile in risk factor subgroup. Adjusted according to model 3. NGT, normal glucose tolerance; IFG, impaired fasting glucose; CKD, chronic kidney disease; ESRD, end-stage renal disease; remnant-C, remnant-cholesterol

Subgroup analyses based on risk factors for ESRD were performed, as described in Fig. 2 and Supplemental Table 4. All subgroups exhibited an increasing risk for incident ESRD as remnant C levels increased. A stepwise escalation of ESRD risk with increasing levels of remnant-C was maintained in those without CKD, who are less susceptible to ESRD than those with underlying renal dysfunction. The increased risk for developing ESRD associated with remnant-C was significantly higher in subjects with dyslipidemia (*P* for interaction < 0.001). Participants undergoing statin treatment also exhibited a stronger relationship between remnant-C and ESRD than those not taking statins, which may be attributed to the greater prevalence of dyslipidemia among statin users (*P* for interaction < 0.001). In contrast, fibrate use had no significant effect on the association, possibly because of the wide confidence intervals from the limited number of fibrate users. Interestingly, the association tended to be attenuated in individuals with hypertension (*P* = 0.052) and weakened in those with hyperglycemia (*P* < 0.001) compared to that in their healthy counterparts.

## Discussion

The nationwide, population-based cohort study found a significant association between elevated remnant-C levels and progression to ESRD over more than 10 years of follow-up. Moreover, this association persisted even after adjusting for conventional risk factors for ESRD, lipid-lowering agents, and total cholesterol levels, demonstrating the independent contribution of remnant-C to incident ESRD. Individuals in the top quartile of remnant-C had a 1.6 times greater risk for incident ESRD compared with those in the lowest quartile in the fully adjusted model. The association was more evident in younger age groups and adult females, with a > 10-fold increased risk in the highest compared to the lowest quartile in females in their 20 s. Subgroup analyses confirmed the persistent pattern of higher ESRD incidence in individuals with higher remnant-C levels across all age, sex, and comorbidity groups.

Accumulating data have strongly supported a causal role of dyslipidemia in both atherosclerosis and nephropathy [38]. Proposed mechanisms include not only the aggravation of renovascular atherosclerosis but also glomerulosclerosis and tubulointerstitial fibrosis caused by oxidative stress, inflammation, downregulation of vasodilators and growth inhibitory cytokines, and glomerular lipid deposition [39]. However, despite this theoretical background, clinical studies have reported inconsistent results regarding the predictive ability of cholesterol or TGs for CKD, leading to uncertainty regarding which component of the lipid profile significantly contributes to CKD progression [7–11, 40]. In this context, recent evidence suggests that the impact of remnant-C on vascular disease extends beyond its traditional lipid component [13–17]. Remnant-C has been recognized as a significant contributor to the residual risk for cardiovascular disease, even when LDL-C levels are well controlled [14]. Considering the overlapping pathogenesis of microvascular and macrovascular disease, further evaluation of the relationship between remnant-C and ESRD is needed.

The relationship between remnant-C and renal dysfunction, independent of conventional lipid parameters, has been suggested by some previous cross-sectional studies [18, 19]. For example, after adjusting for HDL-C and LDL-C levels, a study involving 7356 Chinese individuals revealed a 1.3-fold higher prevalence of CKD in the fourth quartile of calculated remnant-C levels than in the first quartile [18]. Moreover, in 395 individuals without DM, those with CKD exhibited a higher cholesterol content in VLDL and IDL—commonly referred to as remnant-C—while their cholesterol contents in LDL and HDL were lower than those in controls without CKD [19]. Several longitudinal studies have also found an increased risk for diabetic nephropathy increases with a higher baseline remnant-C levels; however, these were limited only to individuals with DM and were relatively small studies with 4000 to 5000 subjects [21, 22]. Nevertheless, to date, there is insufficient supportive evidence to establish a clear relationship between remnant-C and the development of ESRD on a population basis. In this regard, this study represents the first population-based longitudinal investigation of the connection between remnant-C and ESRD development, with the majority (91.5%) of the total population comprising individuals without DM. Importantly, this study found that the effect of remnant-C on incident ESRD was more pronounced in individuals with glucose tolerance than in those with IFG or DM. These findings highlight the broader implications of remnant-C in the development of renal dysfunction, making it an integral factor in the context of overall kidney health and not limited to DM-related conditions.

Consistent with previous studies, the current study demonstrated that conventional risk factors associated with kidney disease, such as older age, male sex, elevated blood pressure, higher BMI, and the presence of DM, were more common in individuals with elevated levels of remnant-C [13]. Of interest, the detrimental effect of remnant-C on the development of ESRD was more pronounced in low-risk groups. More specifically, individuals in their 20 s and 30 s exhibited 4.1- and 2.4-times higher risks for ESRD in the fourth quartile, respectively, whereas those ≥ 70 years of age had a 1.3 times higher risk. Similarly, increased susceptibility to ESRD due to higher remnant-C levels was more evident in females, those with normal blood pressure, and those with glucose tolerance than in males, individuals with hypertension, and those with impaired glucose tolerance. This finding highlights the previously unrecognized significance of elevated remnant-C levels in assessing the risk for ESRD in the general population, and its importance is even more pronounced in individuals with a lower risk for kidney disease. A prospective study involving 4237 individuals with type 2 DM reported no difference in the risk for incident diabetic nephropathy associated with baseline and cumulative levels of remnant-C across risk factor subgroups, including age, sex, and fasting glucose levels [22]. On the other hand, this large study involving > 3.8 million participants was able to identify those with elevated levels of remnant-C who were at higher risk for developing ESRD. Meanwhile, the effect of remnant-C on progression to ESRD was stronger in individuals with dyslipidemia, suggesting a potential synergistic effect between remnant-C and other components of dyslipidemia on the risk for ESRD, which warrants further investigation.

However, the underlying mechanism linking remnant-C to the pathophysiology of ESRD remains unclear. In the context of atherogenic dyslipidemia, which is prevalent among individuals with renal dysfunction, overproduction and inadequate catabolism of TRLs results in increased formation of remnant TRLs and remnant-C [14]. Remnant TRLs produced through partial lipolysis are able to directly penetrate the arterial endothelium via active transcytosis; consequently, remnant-C within these remnant TRLs is retained in subendothelial lesions, triggering the initiation and exacerbation of vascular disease [28]. At the endothelial surface, the TG component in remnant TRLs can be degraded through lipolysis by lipoprotein lipase, releasing large amounts of toxic fatty acids [41]. This process eventually promotes the production of proinflammatory cytokines and atherogenic adhesion molecules [41]. Whether remnant-C is a pathological cause of renal dysfunction or a bystander in nephropathy requires further mechanistic elucidation. Nevertheless, the current finding that high remnant-C was independently associated with incident ESRD—together with robust evidence that remnant-C plays an important role in atherosclerosis, endothelial damage, and inflammation—suggests that remnant-C impairs renal function. Notably, remnant-C-related nephropathy may develop prematurely in apparently healthy individuals with normal renal function because high remnant-C levels have been shown to increase the risk for ESRD, even in individuals without dyslipidemia or CKD.

### Strengths and limitations

Results of this study provide novel insights into the association between remnant-C and ESRD on a population basis with a robust national cohort with > 10 years’ follow-up. However, the present study had several limitations. First, because of the observational design of the study, causality could not be established. Second, calculated rather than measured levels of remnant-C and LDL-C were used, which raises concerns of error from actual levels. To increase the reliability of LDL-C estimation, individuals with very high TG levels were excluded and the Martin-Hopkins equation was used to compensate for the varying ratio of cholesterol to TG in TRLs [42]. In addition, calculated remnant-C has been shown to be a more economical and accessible estimate of remnant-C for clinical use [12]. Direct measurement of remnant-C is costly and labor intensive, making its use in nationwide studies challenging [42]. Both calculated and measured remnant-C confer a similar risk of atherosclerosis at the population level, although measured levels may identify 5% more of those at increased risk of ischemic heart disease at the individual level [43]. Third, the results observed in the Korean population may not be generalizable to all ethnic groups. Nevertheless, the large size of this study raises the possibility that the current findings may be applicable to a broader population. Future research will need to validate the current findings across different ethnicities. Fourth, the definition of dyslipidemia based on total cholesterol levels or prescription of lipid-lowering agents may not be fully consistent with international guidelines [44]; however, this definition has been adopted and established in numerous studies using the Korean NHIS database [16, 31, 32]. Fifth, the potential impact of clinical changes during follow-up was not examined because data were collected only at baseline. Sixth, the possible effect of medications other than statins or fibrates that may have influenced the association between remnant-C and ESRD was not controlled. Finally, the identification of morbidities and prescriptions relied on ICD-10 codes that are subject to potential misclassification. Moreover, the lifestyle and economic information obtained through self-reports or medical records may have been inaccurate.

## Conclusions

The Korean nationwide cohort study demonstrated that higher levels of remnant-C were associated with progression to ESRD, independent of conventional risk factors. The stepwise relationship between remnant-C and ESRD was consistently observed in all subgroups and was more prominent in low-risk groups such as younger patients and adult females. This finding suggests a previously unknown role of remnant-C in predicting ESRD, regardless of the underlying risk conditions. Remnant-C may be the answer to the unidentified triggers of renal dysfunction associated with lipid disorders. In addition, these findings may serve as a framework for the use of remnant-C measurements in risk stratification for ESRD.

### Supplementary Information

**Additional file 1: Supplemental Fig. 1.** Flowchart of study participants. **Supplemental Table 1.** Definitions of outcomes and comorbidities. **Supplemental Table 2.** Risk for ESRD across quartiles of each lipid parameter. **Supplemental Table 3.** Risk for ESRD according to remnant-C quartile in age subgroup for each sex. **Supplemental Table 4.** Risk for ESRD according to remnant-C quartile in risk factor subgroup.

## Data Availability

The datasets analyzed during the current study are available in the National Health Insurance Sharing Service repository, [https://nhiss.nhis.or.kr/bd/ay/bdaya001iv.do].

## Abbreviations

Remnant-C: Remnant-cholesterol
ESRD: End-stage renal disease
HR: Hazard ratio
CI: Confidence interval
CKD: Chronic kidney disease
HDL-C: High-density lipoprotein cholesterol
TG: Triglyceride
IDL: Intermediate-density lipoprotein
VLDL: Very-low-density lipoprotein
LDL-C: Low-density lipoprotein cholesterol
TRL: Triglyceride-rich lipoprotein
DM: Diabetes mellitus
NHIS: National Health Insurance Service
BMI: Body mass index
VLDL-C: Very-low-density lipoprotein cholesterol
ICD-10: International Classification of Diseases, 10th Revision
aHR: Adjusted hazard ratio
IFG: Impaired fasting glucose
