# Supplementary material for: High remnant-cholesterol levels increase the risk for end-stage renal disease: a nationwide, population-based, cohort study
Source: Lipids Health Dis. 2024 Jun 4;23:165. doi: 10.1186/s12944-024-02050-y (PMC11149355; doi:10.1186/s12944-024-02050-y)
Supplement: Supplementary file 1 — Additional file 1: Supplemental Fig. 1. Flowchart of study participants. Supplemental Table 1. Definitions of outcomes and comorbidities. Supplemental Table 2. Risk for ESRD across quartiles of each lipid parameter. Supplemental Table 3. Risk for ESRD according to remnant-C quartile in age subgroup for each sex. Supplemental Table 4. Risk for ESRD according to remnant-C quartile in risk factor subgroup. [file 12944_2024_2050_MOESM1_ESM.doc]

High remnant-cholesterol levels increase the risk for end-stage renal disease:

a nationwide, population-based, cohort study

Supplemental Material

Contents

**Supplemental Fig. 1** Flowchart of study participants

**Supplemental Table 1.** Definitions of outcomes and comorbidities

**Supplemental Table 2.** Risk for ESRD across quartiles of each lipid parameter

**Supplemental Table 3.** Risk for ESRD according to remnant-C quartile in age subgroup for each sex

**Supplemental Table 4.** Risk for ESRD according to remnant-C quartile in risk factor subgroup


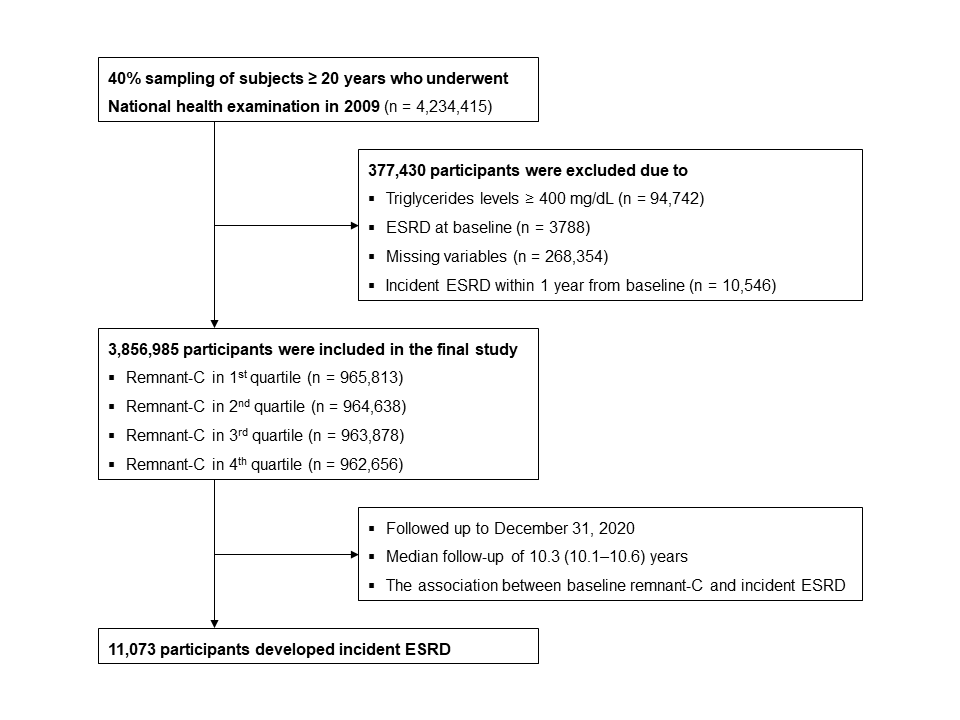


**Supplemental Fig. 1** Flowchart of study participants

ESRD, end-stage renal disease; remnant-C, remnant-cholesterol

**Supplemental Table 1.** Definitions of outcomes and comorbidities

|  | **ICD-10-CM codes** | **Diagnostic definition** |
| --- | --- | --- |
| **ESRD** | V001, V003, V005 | Hemodialysis, peritoneal dialysis, or kidney transplantation |
| **Hypertension** | I10–I15 | Blood pressure ≥ 140/90 mmHg or prescription for antihypertensive medication |
| **DM** | E11–14 | ICD-10 codes for DM with use of antidiabetic medications or fasting blood glucose levels ≥ 126 mg/dL |
| **Dyslipidemia** | E78 | Total cholesterol ≥ 240 mg/dL or use of lipid-lowering agents |
| **CKD** | - | eGFR < 60 mL/min/1.73 m^2^ |
| **Obesity** | - | BMI ≥ 25 kg/m^2^ according to the World Health Organization Asia-Pacific criteria |

ICD-10-CM, International Classification of Diseases, Tenth Edition, Clinical Modification; ESRD, end-stage renal disease; DM, diabetes mellitus; CKD, chronic kidney disease; eGFR, estimated glomerular filtration rate; BMI, body mass index.

**Supplemental Table 2.** Risk for ESRD across quartiles of each lipid parameter

| Subgroup | Quartile group | Number | Events | Duration  (person-years) | Incident rate  (per 1000 person-years) | HRs (95% CI) |
| --- | --- | --- | --- | --- | --- | --- |
| Remnant cholesterol | Q1 | 965,813 | 1239 | 9,823,491 | 0.13 | Reference |
|  | Q2 | 964,638 | 2298 | 9,748,549 | 0.24 | 1.21 (1.13–1.30) |
|  | Q3 | 963,878 | 3079 | 9,719,398 | 0.32 | 1.28 (1.20–1.37) |
|  | Q4 | 962,656 | 4457 | 9,707,940 | 0.46 | 1.56 (1.47–1.67) |
| Total cholesterol | Q1 | 981,459 | 3308 | 9,872,237 | 0.34 | Reference |
|  | Q2 | 933,259 | 2289 | 9,451,143 | 0.24 | 0.89 (0.84–0.94) |
|  | Q3 | 969,976 | 2351 | 9,831,709 | 0.24 | 0.87 (0.82–0.92) |
|  | Q4 | 972,291 | 3125 | 9,844,289 | 0.32 | 0.98 (0.94–1.03) |
| HDL cholesterol | Q1 | 986,502 | 4745 | 9,902,599 | 0.48 | Reference |
|  | Q2 | 942,724 | 2510 | 9,542,709 | 0.26 | 0.73 (0.69–0.76) |
|  | Q3 | 992,296 | 2089 | 10,064,825 | 0.21 | 0.69 (0.65–0.72) |
|  | Q4 | 935,463 | 1729 | 9,489,246 | 0.18 | 0.72 (0.68–0.76) |
| LDL cholesterol | Q1 | 944,230 | 3281 | 9,496,283 | 0.35 | Reference |
|  | Q2 | 976,642 | 2490 | 9,889,558 | 0.25 | 0.95 (0.90–1.00)^a^ |
|  | Q3 | 974,525 | 2366 | 9,876,529 | 0.24 | 0.90 (0.85–0.95) |
|  | Q4 | 961,588 | 2936 | 9,737,008 | 0.30 | 0.98 (0.93–1.03) |
| Triglyceride | Q1 | 976,208 | 1225 | 9,934,252 | 0.12 | Reference |
|  | Q2 | 953,840 | 2189 | 9,643,964 | 0.23 | 1.21 (1.13–1.30) |
|  | Q3 | 965,439 | 3132 | 9,731,323 | 0.32 | 1.32 (1.23–1.41) |
|  | Q4 | 961,498 | 4527 | 9,689,839 | 0.47 | 1.58 (1.48–1.69) |

Adjusted according to model 3.

^a^The upper bound of the 95% CI was 0.999.

ESRD, end-stage renal disease; HR, hazard ratio; CI, confidence interval; HDL, high-density lipoprotein; LDL, low-density lipoprotein.

**Supplemental Table 3.** Risk for ESRD according to remnant-C quartile in age subgroup for each sex

|  |  | Men | | | | | Women | | | | |
| --- | --- | --- | --- | --- | --- | --- | --- | --- | --- | --- | --- |
| Age, years | Remnant-C | N | Events | Duration  (person-years) | Incident rate  (per 1000 person-years) | HRs (95% CI) | Number | Events | Duration  (person-years) | Incident rate  (per 1000 person-years) | HRs (95% CI) |
| Total | Q1 | 368,918 | 810 | 3,697,717 | 0.22 | Reference | 596,895 | 429 | 6,125,774 | 0.07 | Reference |
|  | Q2 | 486,288 | 1,418 | 4,869,292 | 0.29 | 1.11 (1.02–1.21) | 478,350 | 880 | 4,879,257 | 0.18 | 1.43 (1.28–1.61) |
|  | Q3 | 567,208 | 1,908 | 5,695,708 | 0.33 | 1.17 (1.08–1.28) | 396,670 | 1,171 | 4,023,691 | 0.29 | 1.55 (1.38–1.73) |
|  | Q4 | 658,432 | 2,861 | 6,639,686 | 0.43 | 1.42 (1.31–1.55) | 304,224 | 1,596 | 3,068,255 | 0.52 | 1.99 (1.78–2.23) |
| 20–29 | Q1 | 72,070 | 17 | 742,207 | 0.02 | Reference | 144,184 | 36 | 1,486,487 | 0.02 | Reference |
|  | Q2 | 63,929 | 25 | 658,282 | 0.04 | 1.70 (0.92–3.15) | 55,435 | 29 | 571,035 | 0.05 | 2.45 (1.50–4.00) |
|  | Q3 | 53,851 | 31 | 554,524 | 0.06 | 2.52 (1.40–4.56) | 21,462 | 12 | 220,885 | 0.05 | 2.51 (1.30–4.83) |
|  | Q4 | 44,704 | 52 | 459,648 | 0.11 | 4.88 (2.82–8.46) | 8456 | 19 | 86,859 | 0.22 | 10.13 (5.79–17.72) |
| 30–39 | Q1 | 90,479 | 35 | 930,218 | 0.04 | Reference | 105,512 | 34 | 1,085,450 | 0.03 | Reference |
|  | Q2 | 120,558 | 92 | 1,239,667 | 0.07 | 1.93 (1.31–2.85) | 56,087 | 37 | 576,715 | 0.06 | 2.02 (1.27–3.22) |
|  | Q3 | 140,635 | 100 | 1,446,343 | 0.07 | 1.70 (1.16–2.50) | 28,723 | 25 | 294,944 | 0.09 | 2.52 (1.50–4.22) |
|  | Q4 | 174,131 | 223 | 1,788,604 | 0.13 | 2.64 (1.84–3.78) | 14,495 | 34 | 148,796 | 0.23 | 5.87 (3.64–9.48) |
| 40–49 | Q1 | 76,117 | 124 | 777,915 | 0.16 | Reference | 188,175 | 79 | 1,938,563 | 0.04 | Reference |
|  | Q2 | 112,700 | 195 | 1,151,668 | 0.17 | 0.95 (0.76–1.19) | 143,704 | 139 | 1,480,131 | 0.09 | 2.04 (1.55–2.69) |
|  | Q3 | 147,167 | 275 | 1,505,528 | 0.18 | 0.94 (0.76–1.16) | 97,219 | 139 | 1,000,780 | 0.14 | 2.59 (1.96–3.42) |
|  | Q4 | 196,607 | 521 | 2,010,717 | 0.26 | 1.12 (0.92–1.37) | 56,527 | 152 | 580,724 | 0.26 | 3.67 (2.78–4.84) |
| 50–59 | Q1 | 58,995 | 180 | 594,250 | 0.30 | Reference | 97,689 | 85 | 1,007,538 | 0.08 | Reference |
|  | Q2 | 89,171 | 278 | 900,050 | 0.31 | 0.93 (0.77–1.12) | 115,474 | 184 | 1,190,959 | 0.16 | 1.59 (1.23–2.05) |
|  | Q3 | 114,107 | 409 | 1,154,836 | 0.35 | 0.98 (0.83–1.17) | 113,268 | 239 | 1,167,939 | 0.21 | 1.77 (1.38–2.28) |
|  | Q4 | 139,460 | 737 | 1,411,141 | 0.52 | 1.26 (1.06–1.48) | 94,324 | 354 | 971,004 | 0.37 | 2.59 (2.03–3.29) |
| 60–69 | Q1 | 44,879 | 247 | 434,845 | 0.57 | Reference | 42,317 | 111 | 431,812 | 0.26 | Reference |
|  | Q2 | 63,674 | 416 | 618,886 | 0.67 | 1.01 (0.86–1.18) | 68,588 | 254 | 699,735 | 0.36 | 1.22 (0.98–1.53) |
|  | Q3 | 74,011 | 630 | 721,525 | 0.87 | 1.18 (1.02–1.37) | 83,165 | 396 | 847,954 | 0.47 | 1.41 (1.14–1.74) |
|  | Q4 | 73,179 | 834 | 713,436 | 1.17 | 1.39 (1.20–1.61) | 78,848 | 590 | 802,526 | 0.74 | 1.91 (1.56–2.35) |
| ≥70 | Q1 | 26,378 | 207 | 218,283 | 0.95 | Reference | 19,018 | 84 | 175,923 | 0.48 | Reference |
|  | Q2 | 36,256 | 412 | 300,738 | 1.37 | 1.22 (1.04–1.45) | 39,062 | 237 | 360,681 | 0.66 | 1.21 (0.94–1.55) |
|  | Q3 | 37,437 | 463 | 312,952 | 1.48 | 1.23 (1.04–1.45) | 52,833 | 360 | 491,188 | 0.73 | 1.27 (1.00–1.62) |
|  | Q4 | 30,351 | 494 | 256,140 | 1.93 | 1.38 (1.17–1.63) | 51,574 | 447 | 478,345 | 0.93 | 1.43 (1.13–1.81) |

Adjusted according to model 3.

ESRD, end-stage renal disease; remnant-C, remnant-cholesterol; HR, hazard ratio; CI, confidence interval.

**Supplemental Table 4.** Risk for ESRD according to remnant-C quartile in risk factor subgroup

| Subgroup | Remnant-C | Number | Events | Duration  (person-years) | Incident rate  (per 1000 person-years) | HRs (95% CI) | *P* for interaction |
| --- | --- | --- | --- | --- | --- | --- | --- |
| Hypertension (-) | Q1 | 830,464 | 436 | 8,493,710 | 0.05 | Reference | 0.052 |
|  | Q2 | 744,351 | 597 | 7,588,351 | 0.08 | 1.26 (1.11–1.43) |  |
|  | Q3 | 678,457 | 649 | 6,911,628 | 0.09 | 1.33 (1.18–1.50) |  |
|  | Q4 | 623,520 | 903 | 6,356,010 | 0.14 | 1.81 (1.61–2.04) |  |
| Hypertension (+) | Q1 | 135,349 | 803 | 1,329,781 | 0.60 | Reference |  |
|  | Q2 | 220,287 | 1,701 | 2,160,197 | 0.79 | 1.19 (1.10–1.30) |  |
|  | Q3 | 285,421 | 2,430 | 2,807,770 | 0.87 | 1.27 (1.17–1.38) |  |
|  | Q4 | 339,136 | 3,554 | 3,351,930 | 1.06 | 1.54 (1.42–1.67) |  |
| Normal | Q1 | 776,037 | 550 | 7,927,875 | 0.07 | Reference | < 0.001 |
|  | Q2 | 696,035 | 968 | 7,079,213 | 0.14 | 1.44 (1.30–1.60) |  |
|  | Q3 | 631,187 | 1,055 | 6,410,380 | 0.16 | 1.46 (1.31–1.62) |  |
|  | Q4 | 554,530 | 1,229 | 5,637,066 | 0.22 | 1.76 (1.58–1.95) |  |
| IFG | Q1 | 151,335 | 186 | 1,528,406 | 0.12 | Reference |  |
|  | Q2 | 202,375 | 366 | 2,036,157 | 0.18 | 1.18 (0.99–1.41) |  |
|  | Q3 | 239,363 | 488 | 2,410,898 | 0.20 | 1.21 (1.02–1.43) |  |
|  | Q4 | 278,550 | 696 | 2,813,368 | 0.25 | 1.44 (1.22–1.70) |  |
| Diabetes mellitus | Q1 | 38,441 | 503 | 367,210 | 1.37 | Reference |  |
|  | Q2 | 66,228 | 964 | 633,179 | 1.52 | 1.03 (0.93–1.15) |  |
|  | Q3 | 93,328 | 1,536 | 898,120 | 1.71 | 1.18 (1.07–1.31) |  |
|  | Q4 | 129,576 | 2,532 | 1,257,506 | 2.01 | 1.51 (1.37–1.67) |  |
| Obesity (-) | Q1 | 818,270 | 962 | 8,325,344 | 0.12 | Reference | 0.666 |
|  | Q2 | 711,824 | 1,540 | 7,188,093 | 0.21 | 1.21 (1.11–1.31) |  |
|  | Q3 | 607,859 | 1,849 | 6,113,156 | 0.30 | 1.30 (1.20–1.41) |  |
|  | Q4 | 482,495 | 2,231 | 4,842,693 | 0.46 | 1.58 (1.46–1.71) |  |
| Obesity (+) | Q1 | 147,543 | 277 | 1,498,147 | 0.18 | Reference |  |
|  | Q2 | 252,814 | 758 | 2,560,456 | 0.30 | 1.25 (1.09–1.44) |  |
|  | Q3 | 356,019 | 1,230 | 3,606,243 | 0.34 | 1.31 (1.15–1.49) |  |
|  | Q4 | 480,161 | 2,226 | 4,865,247 | 0.46 | 1.67 (1.47–1.89) |  |
| CKD (-) | Q1 | 916,386 | 701 | 9,331,642 | 0.08 | Reference | 0.002 |
|  | Q2 | 902,455 | 1,128 | 9,144,075 | 0.12 | 1.16 (1.05–1.27) |  |
|  | Q3 | 891,848 | 1,324 | 9,025,655 | 0.15 | 1.12 (1.02–1.23) |  |
|  | Q4 | 884,074 | 1,987 | 8,954,062 | 0.22 | 1.46 (1.33–1.59) |  |
| CKD (+) | Q1 | 49,427 | 538 | 491,849 | 1.09 | Reference |  |
|  | Q2 | 62,183 | 1,170 | 604,473 | 1.94 | 1.26 (1.14–1.40) |  |
|  | Q3 | 72,030 | 1,755 | 693,743 | 2.53 | 1.43 (1.29–1.57) |  |
|  | Q4 | 78,582 | 2,470 | 753,878 | 3.28 | 1.64 (1.49–1.81) |  |
| Dyslipidemia (-) | Q1 | 904,450 | 886 | 9,208,318 | 0.10 | Reference | < 0.001 |
|  | Q2 | 841,049 | 1,463 | 8,510,838 | 0.17 | 1.25 (1.15–1.36) |  |
|  | Q3 | 780,563 | 1,790 | 7,882,566 | 0.23 | 1.30 (1.20–1.41) |  |
|  | Q4 | 654,440 | 1,950 | 6,614,435 | 0.29 | 1.44 (1.32–1.56) |  |
| Dyslipidemia (+) | Q1 | 61,363 | 353 | 615,173 | 0.57 | Reference |  |
|  | Q2 | 123,589 | 835 | 1,237,711 | 0.67 | 1.22 (1.07–1.38) |  |
|  | Q3 | 183,315 | 1,289 | 1,836,832 | 0.70 | 1.38 (1.22–1.55) |  |
|  | Q4 | 308,216 | 2,507 | 3,093,505 | 0.81 | 1.92 (1.71–2.16) |  |
| Statin (-) | Q1 | 925,261 | 914 | 9,420,744 | 0.10 | Reference | < 0.001 |
|  | Q2 | 889,224 | 1,580 | 8,999,961 | 0.18 | 1.25 (1.15–1.36) |  |
|  | Q3 | 863,784 | 2,019 | 8,724,070 | 0.23 | 1.30 (1.20–1.41) |  |
|  | Q4 | 833,423 | 2,703 | 8,418,559 | 0.32 | 1.53 (1.41–1.66) |  |
| Statin (+) | Q1 | 40,552 | 325 | 402,747 | 0.81 | Reference |  |
|  | Q2 | 75,414 | 718 | 748,587 | 0.96 | 1.16 (1.02–1.32) |  |
|  | Q3 | 100,094 | 1,060 | 995,328 | 1.06 | 1.31 (1.15–1.48) |  |
|  | Q4 | 129,233 | 1,754 | 1,289,381 | 1.36 | 1.78 (1.58–2.01) |  |
| Fibrate (-) | Q1 | 963,214 | 1214 | 9,797,663 | 0.12 | Reference | 0.427 |
|  | Q2 | 959,938 | 2225 | 9,702,079 | 0.23 | 1.21 (1.13–1.30) |  |
|  | Q3 | 956,485 | 2972 | 9,645,906 | 0.31 | 1.30 (1.21–1.39) |  |
|  | Q4 | 945,185 | 4236 | 9,532,942 | 0.44 | 1.61 (1.50–1.73) |  |
| Fibrate (+) | Q1 | 2599 | 25 | 25,828 | 0.97 | Reference |  |
|  | Q2 | 4700 | 73 | 46,470 | 1.57 | 1.50 (0.95–2.36) |  |
|  | Q3 | 7393 | 107 | 73,493 | 1.46 | 1.42 (0.92–2.19) |  |
|  | Q4 | 17,471 | 221 | 174,998 | 1.26 | 1.60 (1.06–2.42) |  |

Adjusted according to model 3.

ESRD, end-stage renal disease; remnant-C, remnant-cholesterol; HR, hazard ratio; CI, confidence interval; IFG, impaired fasting glucose; CKD, chronic kidney disease.
